# Subjective Sleep Quality and Sleep Architecture in Patients With Migraine: A Meta-analysis

**DOI:** 10.1212/WNL.0000000000012701

**Published:** 2021-10-19

**Authors:** Emily Charlotte Stanyer, Hannah Creeney, Alexander David Nesbitt, Philip Robert Holland, Jan Hoffmann

**Affiliations:** From the Wolfson Centre for Age-Related Diseases (E.C.S., H.C., P.R.H., J.H.), Institute of Psychiatry, Psychology & Neuroscience, King's College London; Department of Neurology (A.D.N.), Guy's and St Thomas NHS Foundation Trust; and NIHR-Wellcome Trust King's Clinical Research Facility/SLaM Biomedical Research Centre (J.H.), King's College Hospital, London, UK.

## Abstract

**Background and Objectives:**

Sleep disturbance is often associated with migraine. However, there is a paucity of research investigating objective and subjective measures of sleep in patients with migraine. This meta-analysis aims to determine whether there are differences in subjective sleep quality measured using the Pittsburgh Sleep Quality Index (PSQI) and objective sleep architecture measured using polysomnography (PSG) between adult and pediatric patients and healthy controls.

**Methods:**

This review was preregistered on PROSPERO (CRD42020209325). A systematic search of 5 databases (Embase, MEDLINE, Global Health, APA PsycINFO, and APA PsycArticles, last searched on December 17, 2020) was conducted to find case–control studies that measured PSG or PSQI in patients with migraine. Pregnant participants and those with other headache disorders were excluded. Effect sizes (Hedges *g*) were entered into a random effects model meta-analysis. Study quality was evaluated with the Newcastle Ottawa Scale and publication bias with the Egger regression test.

**Results:**

Thirty-two studies were eligible, of which 21 measured PSQI or Migraine Disability Assessment Test in adults, 6 measured PSG in adults, and 5 measured PSG in children. The overall mean study quality score was 5/9; this did not moderate any of the results and there was no risk of publication bias. Overall, adults with migraine had higher PSQI scores than healthy controls (*g* = 0.75, *p* < 0.001, 95% confidence interval [CI] 0.54–0.96). This effect was larger in those with a chronic rather than episodic condition (*g* = 1.03, *p* < 0.001, 95% CI 0.37–1.01; *g* = 0.63, *p* < 0.001, 95% CI 0.38–0.88, respectively). For polysomnographic studies, adults and children with migraine displayed a lower percentage of rapid eye movement sleep (*g* = −0.22, *p* = 0.017, 95% CI −0.41 to −0.04; *g* = −0.71, *p* = 0.025, 95% CI −1.34 to −0.10, respectively) than controls. Pediatric patients displayed less total sleep time (*g* = −1.37, *p* = 0.039, 95% CI −2.66 to −0.10), more wake (*g* = 0.52, *p* < 0.001, 95% CI 0.08–0.79), and shorter sleep onset latency (*g* = −0.37, *p* < 0.001, 95% CI −0.54 to −0.21) than controls.

**Discussion:**

People with migraine have significantly poorer subjective sleep quality and altered sleep architecture compared to healthy individuals. Further longitudinal empirical studies are required to enhance our understanding of this relationship.

It has long been recognized that there is a relationship between sleep and migraine. This relationship is complex, as alterations in sleep can be a trigger, treatment, or symptom of migraine.^1^ Whereas there is evidence from both preclinical and prospective clinical research^2-4^ that links sleep with migraine, as well as molecular evidence highlighting the role of orexinergic pathways and melatonin treatment for migraine,^5^ there remains a poor understanding of sleep in migraine. There are an estimated 1 billion people with migraine globally, and migraine is one of the leading causes of disability worldwide,^6^ with a considerable personal and socioeconomic burden.^7^ To reduce this and meet the growing clinical need,^8^ a clearer understanding of the profile of sleep in migraine and its relation to migraine-related disability is important, to enable clinicians to support those with migraine and deliver effective sleep interventions.^9^

Despite its association, there remains a paucity of research into sleep in migraine, and there is no consensus on whether patients exhibit objective changes in sleep architecture. This is partly due to the small sample sizes of polysomnography (PSG) studies that measure sleep. The current meta-analysis aims to overcome this by aggregating data from multiple studies investigating differences in subjective sleep quality as measured by the Pittsburgh Sleep Quality Index (PSQI)^10^ and objective sleep architecture measured using PSG between patients with migraine and healthy controls. Furthermore, the relationship between sleep quality and migraine-related disability is investigated by combining correlational data between PSQI and Migraine Disability Assessment (MIDAS) scores.^11^

## Review Questions

1. Is there a difference in subjective sleep quality as measured by the PSQI between people with migraine and controls? Is this effect larger in those with chronic migraine?
2. Are there differences in sleep architecture as measured using PSG between people with migraine and controls, in both adult and pediatric patients?
3. Is there a positive correlation between subjective sleep quality in adult patients with migraine and migraine-related disability as measured by PSQI and MIDAS questionnaires?

## Methods

This review followed the Preferred Reporting Items for Systematic Reviews and Meta-analysis (PRISMA) guidelines^12^ and the PRISMA 2020 checklists (eTables 1 and 2 available from Dryad, doi.org/10.5061/dryad.vq83bk3sf). The protocol for this review was preregistered on PROSPERO (CRD42020209325).

### Search Strategy

Two authors (E.C.S. and H.C.) conducted an independent search of relevant databases (Embase [1996–2020], MEDLINE [1996–2020], Global Health [1973–2020], APA PsycINFO [1806–2020], APA PsycArticles Full Text [2020]) from their inception to the current date. Search terms included combinations of migraine, sleep*, PSQI, Pittsburgh Sleep Quality Index, polysomnograph*, PSG, EEG, electroencephalograph*, MIDAS, HIT-6, and MSQ with Boolean operators. The full search strategy is available at Dryad (eTable 3, doi.org/10.5061/dryad.vq83bk3sf). The search was limited to studies published in English and duplicates were removed. Titles and abstracts were independently screened by 2 authors (E.C.S. and H.C.). Studies that were eligible or if eligibility was unclear were submitted to full text review. Relevant studies were also retrieved from reference lists of studies. All full texts were screened for eligibility by 1 author (E.C.S.) and 10% of full texts were selected randomly using a random number generator, which the second reviewer (H.C.) screened. There were no discrepancies between the authors. All studies were examined to ensure they were independent of one another. If full texts were not available, the original authors were contacted, and full texts were requested. The last search date was December 17, 2020.

### Inclusion Criteria

Studies were eligible if they examined sleep quality as assessed by the PSQI in adults or sleep variables using PSG in adult and pediatric patients with migraine and controls. Studies that computed correlations between MIDAS and PSQI scores in people with migraine were also included.

### Exclusion Criteria

Review articles or case studies were not included.

### Population

There were no restrictions on the age of participants in the studies. However, for analysis purposes, adults ≥18 years of age and children <18 years of age were included in separate analyses given that sleep demonstrates age-dependent quantitative differences.^13^ Pregnant participants and participants with other headache disorders (cluster headache, tension-type headache, and medication overuse headache) were excluded. However, a study was included if it reported data that could be extracted that was specific to migraine and no other headache disorders. Due to the limited number of studies in this area, we kept the classification of migraine broad, thus patients with migraine with any diagnosis were included in this analysis: episodic, chronic, migraine without aura (MO), migraine with aura (MA). Although this is not a standard definition in the *International Classification of Headache Disorders 3* (*ICHD-3*), studies that categorized sleep-related migraine or non-sleep-related migraine were included. Studies that categorized patients by the number of migraine days per month and not by episodic or chronic status were included. In such cases, we categorized patients experiencing headache/migraine on ≥15 days per month as chronic and pooled them with other chronic cases. As sleep quality may differ between different frequencies of migraine, episodic and chronic migraine were analyzed as separate subgroups.

### Outcome Measures

The primary outcomes calculated were weighted effect sizes (Hedges *g*) for the difference between people with migraine and controls in global PSQI score and PSG-derived sleep measures. PSG-derived measures included total sleep time (TST) in minutes, sleep efficiency percent, percent wake, percent of TST spent in rapid eye movement (REM) sleep, non-REM (NREM) sleep stage 1 (N1), stage 2 (N2), and stage 3 (N3), and sleep onset latency (SOL) in minutes. To ascertain whether subjective sleep quality is related to migraine disability, the Fisher *z* transformed correlation coefficient between MIDAS and PSQI scores in the migraine population was calculated.

### Data Extraction

Data extraction was performed using an a priori elaborated table in Microsoft Excel. Extraction was completed by 1 author (E.C.S.) and included authors, year of publication, journal, publication type, participant demographics, migraine characterization, mean global PSQI score, PSG-derived sleep variable means, correlation coefficient between MIDAS and PSQI scores, SDs, and group sizes. Where measures were not reported in the publication or the data were not in the correct format for analysis (e.g., medians instead of means), authors were contacted to request data. Studies that did not report data from which the effect sizes could be calculated after contacting the authors or in which authors did not respond were excluded. Further data were extracted that might be potential moderators; for example, design of the study, whether the study excluded those on medication (which may affect the sleep cycle or those with comorbid sleep disorders), and presence of a PSG adaptation night or not. If studies used older sleep scoring criteria to determine NREM sleep stages separately (stages 3 and 4), an average of the means for these 2 stages was computed to be comparable to updated American Academy of Sleep Medicine nomenclature, which defines these singularly as N3. If a study reported PSG sleep stage variables in minutes rather than percentages, these were calculated based on TST.

### Statistical Analysis

Statistical analysis was performed using RStudio and the metafor package.^14^ The weighted effect sizes (Hedges *g*) for each study were calculated using the means, sample sizes (n), and SDs. Effect sizes were interpreted as small (0.2), medium (0.5), or large (0.8)^15^ and visualized using forest plots. Effect sizes were calculated such that a negative Hedges *g* indicated that controls had a higher score on that measure. For the PSQI analysis in adults, studies that investigated chronic cases were pooled with episodic cases for a global analysis of effect size; they were then analyzed in 2 subgroups. PSG-derived variables from pediatric and adult patients were analyzed as 2 separate groups. Effect sizes were computed for each PSG measure.

#### Publication Bias

Publication bias refers to the overinflation of effect sizes due to the tendency for nonsignificant findings to remain unpublished. To assess this, Egger regression test for funnel plot asymmetry was performed.^16^ If the result is significant at *p* < 0.05, this provides evidence of publication bias. However, as the Egger test is prone to producing false-positives with small numbers of studies, publication bias was also assessed by visual inspection of funnel plots. These plot precision measures (standard error) against effect size (Hedges *g*). If the funnel plots are substantially asymmetric, publication bias can be assumed. The Duval and Tweedie trim-and-fill method^17^ was used to assess whether any unpublished studies were missing from the analysis and estimate the adjusted effect size if these were present.

#### Between-Studies Heterogeneity

As meta-analysis typically includes studies of varying designs, it is important to quantify the proportion of between-studies heterogeneity to ensure accurate estimation of effect sizes. Cochran *Q* statistic is used to assess this, and if significant at *p* < 0.05, this indicates variability in effect sizes reported between studies. However, as this test has poor power to detect heterogeneity with few studies, the *I*^2^ statistic was also calculated^18^ (0% = no heterogeneity, 25% = low, 50% = moderate, 75% = high). As heterogeneity was to be expected given the variation in study designs, a random-effects model meta-analysis was employed.

To explore heterogeneity in both the PSQI and PSG analyses, where a significant effect and moderate heterogeneity as indexed by an *I*^*2*^ of >50% were found, additional analyses were conducted by including study characteristics as moderators. Moderator variables were not predefined and were based on a previous meta-analysis^19^: exclusion of participants with sleep disorders (yes/no), exclusion of participants taking drugs that affect the sleep cycle or a medication washout period (yes/no), whether controls were matched for sex and age (yes/no). Where a study did not state whether this was conducted, it was coded as “no.”

#### Study Quality Assessment

The Newcastle-Ottawa Scale^20^ (NOS) (eAppendix 1 at Dryad, doi.org/10.5061/dryad.vq83bk3sf) was used to assess the quality of the case–control and cross-sectional studies included in the meta-analysis. Two authors (E.C.S. and H.C.) independently scored each study using the checklist. Discrepancies in study assessments were resolved through discussion. In the case of discrepancies that could not be resolved, these were resolved by a third reviewer (J.H.), who gave the final decision. Study quality scores out of a maximum of 9 (7–9 high quality, 4–6 high risk of bias, 0–3 very high risk of bias) were included as a moderator variable in subsequent analyses.

### Data Availability

The data are available at Dryad (doi.org/10.5061/dryad.vq83bk3sf).

## Results

### Description of Studies

**Figure 1:**
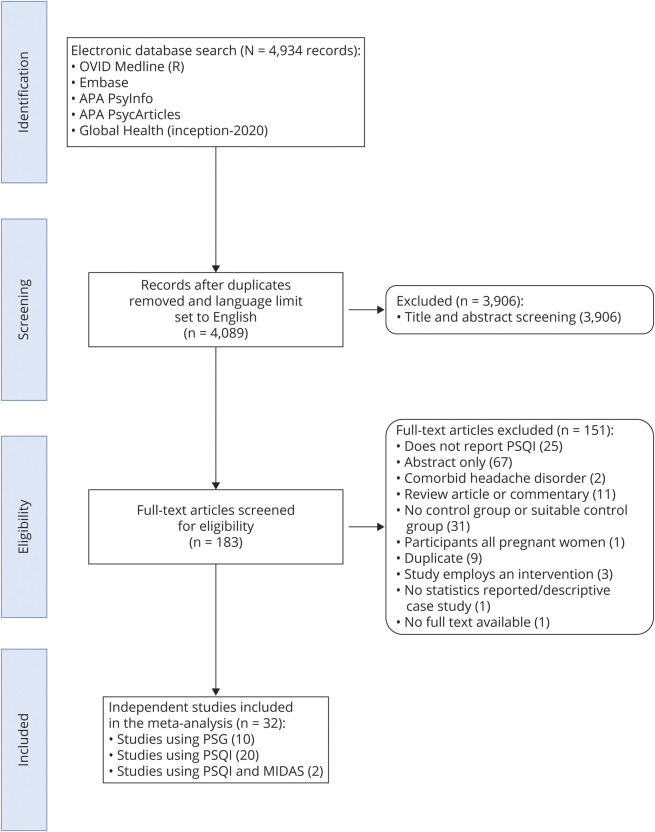
Preferred Reporting Items for Systematic Reviews and Meta-Analyses (PRISMA) Flow Diagram Depicting the Stages of Study Selection MIDAS = Migraine Disability Assessment; PSG = polysomnography; PSQI = Pittsburgh Sleep Quality Index.

Results of the selection process are displayed in Figure 1. From a total of 4,089 studies after duplicates were removed, 183 were identified as potentially eligible after title and abstract screening. Full texts were then screened to confirm this, after which 32 studies (23 case–control and 9 cross-sectional) were included in the meta-analysis. Table 1 presents an overview. Twenty investigated PSQI scores in adults with and without migraine. Of these, 14 of them had a population of episodic migraine and 6 of them chronic migraine. Four studies (2 included in the PSQI analysis) reported the correlation between MIDAS and PSQI scores in those with migraine.^21-24^ Although categorizing patients based on ICHD-3 was not an eligibility requirement, this was assessed during study quality assessment and all studies used these criteria to confirm migraine. There were no participants <18 years of age in the PSQI analysis or the MIDAS and PSQI correlational analysis due to a lack of available data. Eleven studies measured PSG in adults^25-30^ and children.^31-35^ One study^26^ compared PSG from patients with migraine in the preictal, midictal, and postictal phases, and thus we could not extract a single value. However, the study provided a pooled value for the PSQI analysis, thus it was excluded from the PSG analysis only.

**Table 1:**
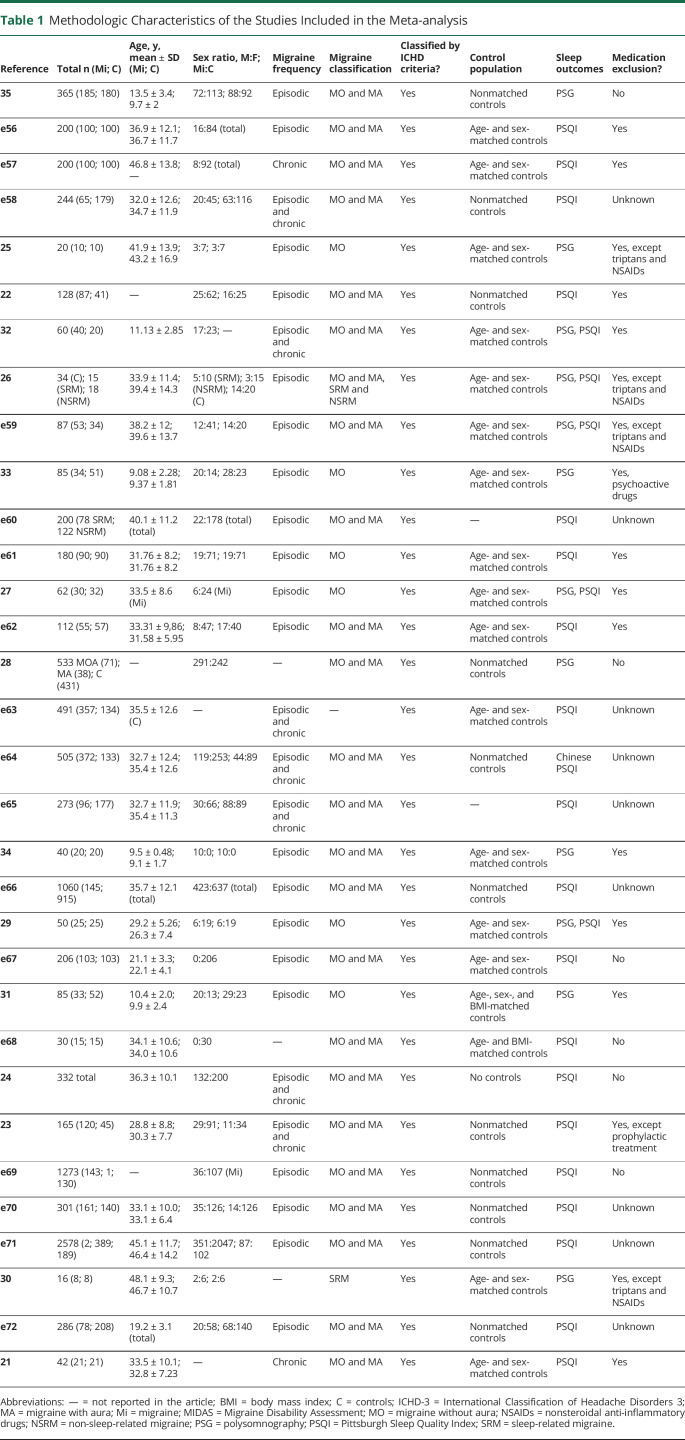
Methodologic Characteristics of the Studies Included in the Meta-analysis

### Risk of Publication Bias Results

There was no indication of publication bias for any of the analyses apart from the correlational analysis as indicated by the Egger test (eTable 4 on Dryad, doi.org/10.5061/dryad.vq83bk3sf). The trim-and-fill method estimated that there was 1 study missing on the right, and this would make the effect size significant but not change the direction of effect (*z* = 0.44, *p* = 0.024). For the PSG analysis, visual inspection of funnel plots, and the trim-and-fill method (eFigure 1 on Dryad, doi.org/10.5061/dryad.vq83bk3sf), revealed that for percentage wake there was likely to be 1 study missing on the right. However, adjustment of effect size did not change the direction of or reduce the effect size, thus publication bias is unlikely to influence the result.

### Study Quality Results

The NOS ratings for each study are included in eTables 5 and 6 (available from Dryad, doi.org/10.5061/dryad.vq83bk3sf). The overall mean score on the NOS was 5 (SD 1.34). The mean score on the NOS was 4.83 (range 2–7, SD 1.43) for case–control studies and 5.44 (range 4–7, SD 0.96) for cross-sectional studies. Four studies were categorized at very high risk of bias, 16 at high risk, and 3 high quality.

### Meta-analysis Results

#### PSQI Score: Adults

**Figure 2:**
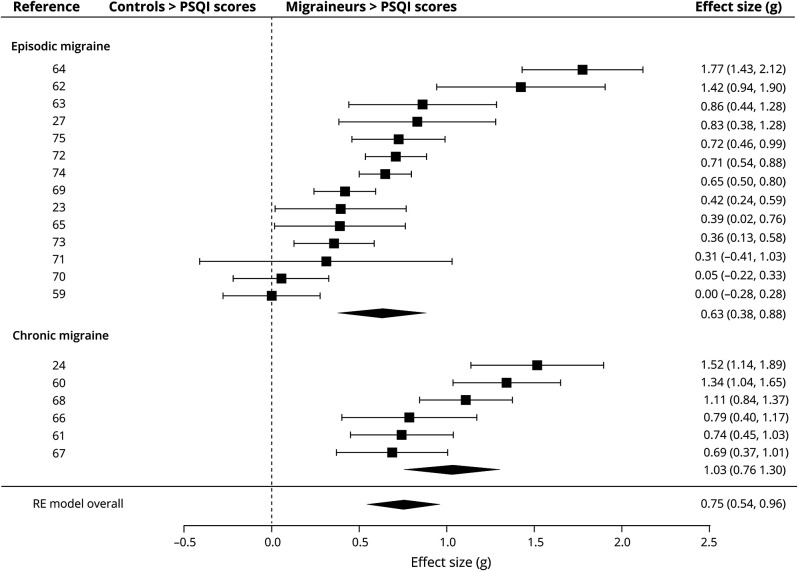
Forest Plot of the Meta-analysis of Pittsburgh Sleep Quality Index (PSQI) Scores in Patients With Migraine and Controls The standardized mean difference (Hedges *g*) and confidence intervals for the difference in global PSQI scores is shown between people with migraine and healthy controls. RE = random effects.

Figure 2 displays the forest plot for the PSQI analysis in adults and controls. There was a medium effect size for the difference in PSQI scores between patients with migraine and controls (*g* = 0.75, *p* < 0.001). There was a large significant effect size for the chronic subgroups (*g* = 1.03, *p* < 0.001) and a medium significant effect size for the episodic subgroup (*g* = 0.63, *p* < 0.001). The direction of these effects indicates that patients scored significantly higher on the PSQI than controls, suggesting poorer sleep quality.

For the overall and episodic analyses, there was statistically significant heterogeneity as evidenced by Cochran *Q* (161.6 and 105.5, respectively). The chronic analysis had significantly lower heterogeneity (20.32). However, when considering the *I*^2^ statistic, the studies within all 3 analyses displayed moderate to high heterogeneity (*I*^*2*^ = 90.8%, 91.6%, 76.7%, respectively), suggesting that over 70% of the variability is attributable to between-study heterogeneity above sampling error.

As there was above moderate heterogeneity, moderator analyses were conducted on the overall analysis (eTable 7 at Dryad, doi.org/10.5061/dryad.vq83bk3sf). There was a negative moderating effect of whether sleep disorders were excluded or not (*Q*_*M*_ = 7.40, *p* = 0.007, *β* = −0.81), suggesting that when sleep disorders were excluded the effect size is smaller. However, there was significant heterogeneity not explained by this moderator (*Q*_*E*_ = 114.61, *p* < 0.001). No other variables were significant moderators.

#### Polysomnography: Adults

Table 2 displays the effect sizes and heterogeneity for the PSG-derived sleep measures. There was a significant small effect size for percentage REM sleep (*g* = −0.22, *p* = 0.017). The direction of this effect indicates that adults with migraine had less REM sleep than controls. There were no significant effect sizes for the other measures. There was significantly moderate heterogeneity as evidenced by the *Q* and *I**^2^* statistic in 2 of the analyses (sleep efficiency and wake). However, as the main effect size for these measures was not significant, no moderator analyses were conducted.

**Table 2:**
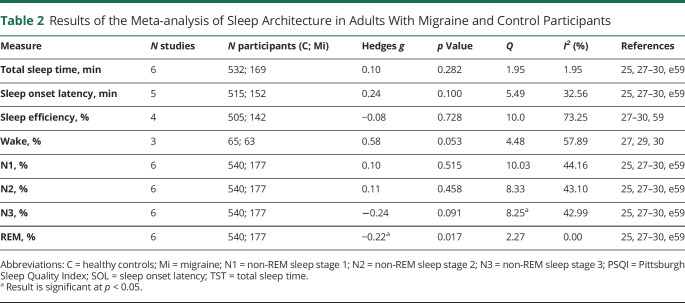
Results of the Meta-analysis of Sleep Architecture in Adults With Migraine and Control Participants

#### Polysomnography: Pediatric

Table 3 displays the effect sizes for the PSG-derived measures in pediatric patients. There were small significant effect sizes for wake (*g* = 0.43, *p* = 0.015) and SOL (*g* = −0.37, *p* < 0.001). There was a medium significant effect size for REM sleep (*g* = −0.71, *p* = 0.025) and a large significant effect size for TST (*g* = −1.37, *p* = 0.039). The direction of these effects indicates that pediatric patients had more wake, less REM sleep, less TST, and shorter SOL than controls. There was statistically significant heterogeneity in 6 analyses (TST, sleep efficiency, N1, N2, N3, REM).

**Table 3:**
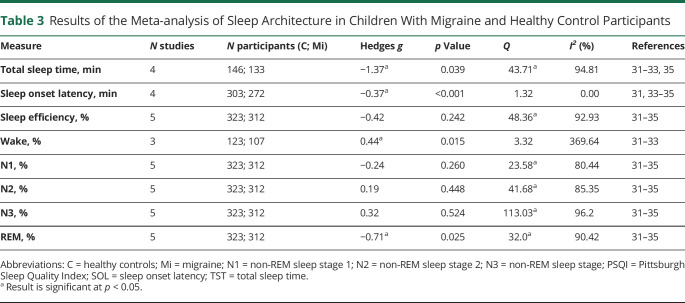
Results of the Meta-analysis of Sleep Architecture in Children With Migraine and Healthy Control Participants

Two of the analyses of PSG variables in pediatric patients that had significant effect sizes and displayed at least moderate heterogeneity were TST and REM; thus a moderator analysis was conducted (eTable 8 on Dryad, doi.org/10.5061/dryad.vq83bk3sf). None of the studies excluded patients with sleep disorders, so this was not included as a moderator. For TST, whether the study excluded patients who were on sleep-affecting medications, and whether the study included matched controls or not, were significant moderators (*Q*_*M*_ = 19.6, *p* < 0.001, *Q*_*M*_ = 19.6, *p* < 0.001, respectively). The test for residual heterogeneity (*Q*_*E*_) was not significant for these analyses, indicating that they are largely influencing the effect size in these studies. Thus, when a study excluded those on medication, or included matched controls, the effect size is reduced. Adaptation night and study quality score were not significant moderators (*Q*_*M*_ = 0.20, *p* = 0.655; Q_*E*_ = 38.46, *p* < 0.001; *Q*_*M*_ = 0.92, *p* = 0.338; Q_*E*_ = 34.99, *p* < 0.001, respectively). For REM sleep, there were no significant moderators.

#### Relationship Between Sleep Quality and Migraine Disability

**Figure 3:**
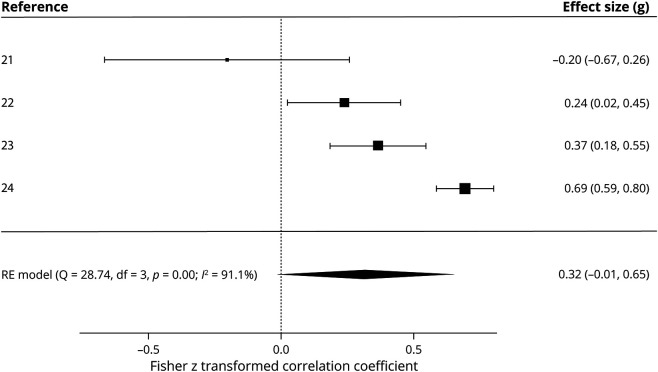
Forest Plot of the Meta-analysis of Migraine Disability Assessment (MIDAS) and Pittsburgh Sleep Quality Index (PSQI) Correlations The Fisher *z* transformed correlation coefficient is shown between MIDAS scores and PSQI scores in patients with migraine across 4 studies. RE = random effects.

Figure 3 shows the effect size for the correlation between MIDAS and PSQI scores. There was a small nonsignificant effect size for the correlation between MIDAS scores and PSQI scores (*z* = 0.32, *p* = 0.060), thus suggesting no relationship between sleep quality and migraine disability. There was significantly low heterogeneity between studies as indicated by the Cochran *Q* statistic (*Q* = 28.74, *p* < 0.001), but *I*^*2*^% indicated high heterogeneity (*I*^*2*^% = 91.1%). As this statistic is more appropriate for small samples, high heterogeneity can be assumed. Because the main analysis was not significant, no moderator analyses were conducted.

## Discussion

This meta-analysis aimed to consolidate previous findings and establish whether subjective sleep quality and objective sleep architecture are altered in migraine. The findings demonstrate that adults with migraine display significantly higher scores on the PSQI, indicating worse subjective sleep quality than in healthy controls, an effect larger in chronic migraine. Sleep architecture is altered; adults and children with migraine display significantly less REM sleep as a percentage of TST than healthy individuals. Pediatric patients also exhibit significantly less TST, shorter SOL, and more wake than controls. There were no differences between patients with migraine and healthy controls in any other sleep measures in either population. Finally, there was no significant overall correlation between PSQI and MIDAS scores in people with migraine.

These findings extend the literature by aggregating the results of multiple studies, thereby increasing power. They demonstrate that sleep quality is worse in adults with migraine than healthy individuals. This finding is not surprising; poor sleep is reported as an exacerbating factor for migraines in 50% of cases,^36^ and the effect size was larger in chronic cases, in line with previous research wherein poor sleep is shown to be an important factor in progression to chronic migraine.^37^

REM sleep was reduced in adults with migraine relative to healthy individuals, aligned with previous associations between migraine and REM sleep.^38^ For example, a reduction in REM sleep has been found the night preceding a migraine attack.^39^ Furthermore, cutaneous allodynia, a prominent symptom during migraine attacks, has been shown to worsen in response to REM sleep deprivation,^3^ thus indicating potential dysfunction in mechanisms underlying REM-NREM or REM-wake transitions in migraine. A plausible neural correlate for this is the hypothalamic orexinergic system, which plays a critical role in stabilizing sleep/wake transitions and REM sleep,^40^ and is linked to migraine, suggesting multiple points of intersection between sleep and trigeminal pain. This is speculative, and the relationship with REM sleep is likely to be complex. There were no differences between adults with migraine and healthy controls in any other measures. This is at odds with a previous meta-analysis that found that TST, wake after sleep onset, and SOL were worse in those with chronic pain than healthy controls.^19^ This might be reconciled in that they had a larger number of studies that included heterogeneous chronic pain conditions. It is likely that with more studies we might see significant differences in other measures.

Pediatric patients also displayed less REM sleep, shorter TST, and more wake, in line with previous findings in children with chronic pain.^41^ Patients with migraine also displayed shorter SOL than controls, suggestive of children with migraine operating at higher sleep pressure due to being chronically sleep deprived (perhaps due to headache, behavior, or both). Alternatively, children with migraine may be biologically sleepier than their healthy counterparts. This is at odds with findings from actigraphy^42^ and questionnaire-based studies^43^ in pediatric patients with migraine, which found longer SOL in patients compared to healthy controls. Actigraphy and self-report data are less accurate than PSG, particularly for SOL.^44^ However, it should be noted that the current effect size is small and generated by a small number of studies.

The lack of a significant difference in NREM sleep in both adults and children with migraine compared to controls is noteworthy, although this only represents macroscopic sleep scoring. Deficits in NREM sleep can be compensated for by an increase in sleep intensity rather than duration, which would not be apparent with macroscopic scoring. These differences could instead be captured by measuring changes in delta power or oscillations during NREM sleep such as sleep spindles.

The lack of a significant correlation between PSQI and MIDAS scores in adults is surprising given that previous studies report significant correlations between migraine-related disability and sleep disturbances,^45^ and altering sleep can have a profound effect on migraine-related disability scores.^46^ This may reflect the small number of studies and high heterogeneity. Three of the studies in this analysis found significant positive correlations,^24^ and 1 found a large negative correlation.^21^ Studies with a small *n* can lead to inflated effect sizes.^47^ Indeed, the study reporting a negative relationship had an *n* of 21,^21^ compared to 332 for one of the larger studies, thus potentially skewing the results towards insignificance. There was also evidence of publication bias within this analysis. Caution should be taken when interpreting the results as despite meta-analysis being theoretically sound on a small number of studies, the Egger test can produce false-positives.

One consideration is that many patients with migraine are given treatments that affect the sleep cycle, and for many studies it was impossible to deduce whether patients on medication were excluded. However, when this was included as a moderator variable in the pediatric analysis for TST, studies that excluded those on medications had a smaller effect size for TST than those that did not, suggesting that medication may be contributing to differences in TST. Indeed, β-blockers—a common migraine preventative—reduce TST.^48^ Nonetheless, significant residual heterogeneity remained that could not be explained by this moderator, suggesting other factors are involved. Medication did not moderate the effect for REM sleep in children.

These results do not provide evidence for a direct relationship between migraine and sleep. Do people with migraine experience poor sleep due to attacks or are they experiencing attacks due to poor sleep? The studies did not report whether patients experienced attacks during sleep itself, despite two-thirds of patients with migraine reporting this.^36^ Only a handful of studies reported whether PSG was conducted in the ictal or interictal period, despite this affecting objective measures of sleep.^49^ If this were to affect the results, however, we might expect a difference in other sleep measures in both populations, yet we only see the decrease in REM sleep, implying specific dysfunction in the mechanisms underlying REM sleep.

Alternatively, reduced REM sleep could reflect that most attacks occur in the early morning where REM sleep dominates, hence curtailing REM opportunity. However, 2 studies included patients with predominantly sleep-related attacks^26,30^ and neither study reported differences in REM sleep between patients and controls. This implies that the reduced REM sleep is not necessarily due to increased arousals during REM.

Moreover, 6 of the studies did not include an adaptation night to the sleep laboratory. The “first night effect” particularly affects measures of REM sleep.^50^ The moderator analyses in children found that adaptation night was not a significant moderator for REM sleep.

The disparity between the large effect size seen with the subjective measure of sleep and the lack of significant difference between patients and controls with all but one of the objective sleep measures in the adult population is not unexpected. Global PSQI scores do not significantly correlate with sleep variables measured via PSG.^e51^ As we did not analyze PSQI scores in the pediatric population, it is unclear whether pediatric patients also experience altered sleep quality. The PSQI has limited utility in pediatrics^e52^ and it is possible that because we only used PSQI as a search term and no other sleep questionnaires, including those used in children, we may have missed studies investigating sleep quality in children. This highlights another limitation, in that other subjective sleep measures such as sleepiness or insomnia indices were not captured by this analysis. Furthermore, unpublished studies were not located due to aiming to include only peer-reviewed, methodologically rigorous studies in this analysis. This means that all the relevant literature on this topic may not have been captured here.

In addition, many studies were retrospective on patients who have previously been referred to sleep clinics with nonspecific sleep complaints, necessitating PSG assessment, suggesting patients may already report underlying sleep complaints. Few of the studies mentioned prior sleep history, and for sleep quality those that did exclude sleep disorders had a smaller effect size than those that did not, suggesting poor sleep quality may be attributable to undiagnosed sleep disorders. However, this is unlikely to be the case as significant residual heterogeneity remained. This heterogeneity may reflect differences in sleep history, recording environments, equipment, and interscorer concordance.^e53^

Many studies did not record whether patients napped before PSG assessment. It is plausible that patients with migraine nap more than controls to relieve their symptoms, meaning less REM during nocturnal sleep. However, if this were the case SOL would be longer in the patients than controls, as recent evidence suggests.^e54^ In addition, mental health was not explored in the current analysis and indeed most studies did not report whether the patients had comorbid mental illness, which may also affect the sleep cycle.

These findings highlight that sleep should play an integrated role in migraine treatment. Clinicians should prioritize sleep interventions and consider sleep when prescribing medication. Indeed, recent studies have demonstrated the utility of sleep interventions in reverting chronic to episodic migraine.^e55^ The relevance of REM sleep and its modulation for migraine and treatment is emphasized by this meta-analysis. The relationship is likely to be complex and related to sleep homeostasis, rather than related to any absolute proportions of REM sleep, a notion supported by the REM-suppressing effects of the best-known migraine preventive, amitriptyline.

People with migraine, particularly those with chronic migraine, report worse subjective sleep quality than healthy individuals. Adults exhibit significantly less REM sleep, whereas children also show significantly reduced sleep time, shorter sleep onset, and more wake than controls. The interplay between migraine and sleep is likely to be complex and remains poorly understood. However, this meta-analysis emphasizes the importance of assessing and treating sleep as an integrated part of migraine treatment. While offering significant insight into sleep in migraine, this study highlights the limitations of drawing conclusions from a small number of case–controlled PSG studies, given the significant confounds and heterogeneity involved. Future studies should analyze the interplay between migraine and subjective sleep on a large scale, as well as use standardized, population-based approaches to sleep architecture to minimize variability.

## Publication History

This manuscript was previously published in MEDRXIV/2021/252791. Received by *Neurology* March 15, 2021. Accepted in final form August 12, 2021.

## Glossary

ICHD-3: International Classification of Headache Disorders 3
MIDAS: Migraine Disability Assessment
NOS: Newcastle-Ottawa Scale
NREM: non-REM
PSG: polysomnography
PRISMA: Preferred Reporting Items for Systematic Reviews and Meta-analysis
PSQI: Pittsburgh Sleep Quality Index
REM: rapid eye movement
SOL: sleep onset latency
TST: total sleep time
